# Niemann-Pick type C1 patient-specific induced pluripotent stem cells display disease specific hallmarks

**DOI:** 10.1186/1750-1172-8-144

**Published:** 2013-09-18

**Authors:** Michaela Trilck, Rayk Hübner, Philip Seibler, Christine Klein, Arndt Rolfs, Moritz J Frech

**Affiliations:** 1 Albrecht-Kossel-Institute for Neuroregeneration (AKos), University of Rostock, Gehlsheimer Strasse 20, D-18147 Rostock, Germany; 2 Institute of Neurogenetics, University of Lübeck, Maria-Goeppert-Strasse 1, 23562 Lübeck, Germany

**Keywords:** Niemann-Pick Type C1, Induced pluripotent stem cells, Lysosomal storage disorder, Neural progenitor cells, Neural differentiation

## Abstract

**Background:**

Niemann-Pick type C1 disease (NPC1) is a rare progressive neurodegenerative disorder caused by mutations in the NPC1 gene. In this lysosomal storage disorder the intracellular transport and sequestration of several lipids like cholesterol is severely impaired, resulting in an accumulation of lipids in late endosomes and lysosomes. The neurological manifestation of the disease is caused by dysfunction and cell death in the central nervous system. Several animal models were used to analyze the impaired pathways. However, the underlying pathogenic mechanisms are still not completely understood and the genetic variability in humans cannot be reflected in these models. Therefore, a human model using patient-specific induced pluripotent stem cells provides a promising approach.

**Methods:**

We reprogrammed human fibroblasts from a NPC1 patient and a healthy control by retroviral transduction with Oct4, Klf4, Sox2 and c-Myc. The obtained human induced pluripotent stem cells (hiPSCs) were characterized by immunocytochemical analyses. Neural progenitor cells were generated and patch clamp recordings were performed for a functional analysis of derived neuronal cells. Filipin stainings and the Amplex Red assay were used to demonstrate and quantify cholesterol accumulation.

**Results:**

The hiPSCs expressed different stem cell markers, e.g. Nanog, Tra-1-81 and SSEA4. Using the embryoid body assay, the cells were differentiated in cells of all three germ layers and induced teratoma in immunodeficient mice, demonstrating their pluripotency. In addition, neural progenitor cells were derived and differentiated into functional neuronal cells. Patch clamp recordings revealed voltage dependent channels, spontaneous action potentials and postsynaptic currents. The accumulation of cholesterol in different tissues is the main hallmark of NPC1. In this study we found an accumulation of cholesterol in fibroblasts of a NPC1 patient, derived hiPSCs, and neural progenitor cells, but not in cells derived from fibroblasts of a healthy individual. These findings were quantified by the Amplex Red assay, demonstrating a significantly elevated cholesterol level in cells derived from fibroblasts of a NPC1 patient.

**Conclusions:**

We generated a neuronal model based on induced pluripotent stem cells derived from patient fibroblasts, providing a human *in vitro* model to study the pathogenic mechanisms of NPC1 disease.

## Background

Human induced pluripotent stem cells (hiPSCs) feature three major advantages in the field of stem cell research. First, cells can be obtained by reprogramming different somatic cells [1-4] without raising ethical concerns, as it is the case with embryonic stem cells. Second, the pluripotent potential of the cells offers the opportunity to differentiate them into each cell of the body, e.g. motor neurons [5], cardiomyocytes [6], pancreatic insulin-producing cells [7], or male germ cells [8]. Third, iPS cells and subsequently differentiated cells have the same genetic information as the donor cells. Different diseases have already been modeled by using human iPS cells, e.g. Parkinson disease [9], metabolic liver disorders [10], retinal degeneration [11], Huntington disease [12], and mucopolysaccharidosis type IIIB, a fatal lysosomal storage disorder [13], and have been successfully utilized e.g. in drug screening [14].

Taken together, these characteristics of the cells are excellent prerequisites to model diseases *in vitro*. However, no *in vitro* model for Niemann-Pick disease Type C1 (NPC1) based on hiPS cells is currently available. NPC1 is a rare progressive neurodegenerative disease caused by mutations in the NPC1 gene located on chromosome 18q11 encoding for a 1278-amino acid intracellular membrane glycoprotein [15-17]. It is inherited in an autosomal recessive manner and shows a prevalence of 1:120.000 live births [18]. A mutation in the NPC1 gene leads to an impaired lipid transport and sequestration resulting in e.g. a cholesterol accumulation in the late endosome and lysosome [19]. The clinical manifestation varies from neonatal icterus and hepatosplenomegaly in early childhood, cerebellar ataxia, seizures, gelastic cataplexy, and vertical supranuclear palsy in adolescence, to progressive neurological degradation, psychoses, and dementia in adulthood [18]. The symptoms are diverse and show intrafamilial variability [18,20].

The pathogenic mechanisms ultimately leading to a massive degeneration and loss of neurons in the CNS, especially Purkinje cells in the cerebellum, are not exactly understood. Most of our knowledge regarding NPC1 is based on cell models like human fibroblasts [21-23] and animal models like mouse [24], cat [25], and fruit fly [26]. Studies using these models have neither revealed the mechanisms leading to the selective massive degeneration of neurons nor found drugs, which can efficiently halt disease progression. Although the function of NPC1 in lipid trafficking is evolutionary highly conserved [27], the widely used murine BALB/c NPC1 model [28] cannot exactly reproduce human pathology. For example, neurofibrillary tangles composed of tau protein, which are seen in human NPC1 neurons, are absent in this model reflecting obvious biochemical and physiological differences [29]. Thus, studies utilizing disease-specific human neurons hold great promise to significantly increase our knowledge in understanding the pathological mechanism leading to massive neuronal degeneration. Recently, a human neuronal NPC1 model was reported based on multipotent adult stem cells [30].

In our study, we generated patient-specific induced pluripotent stem cells from a NPC1 patient and a healthy individual. The hiPS cell lines were differentiated into neural progenitor cells and subsequently differentiated into functional neurons to gain a human neuronal model of NPC1 disease.

## Methods

### Cell culture

Human dermal fibroblast cell lines GM18436 and GM05659 (Coriell Institute for Medical Research, Camden, USA) were obtained by skin biopsies from one-year old male Caucasian donors. GM18436 exhibits compound heterozygous mutations in the NPC1 gene (c.1628delC and GLU612ASP), representing a frameshift mutation and a missense mutation, respectively. The mutations lead to a non-functional protein as demonstrated by cholesterol esterification assay [31]. Fibroblast cell line GM05659 is obtained from a healthy donor. In the following cells of the cell line GM18436 will be referred to as mutNPC1 and cells of the cell line GM05659 will be referred to as wtNPC1. Cells were cultivated in fibroblast medium containing DMEM high glucose, 10% FBS and 1% Penicillin/ Streptomycin. Mitotically inactivated mouse embryonic fibroblasts (GlobalStem, Rockville, USA) were used as the feeder cell layer for hiPSCs. Cells were plated in fibroblast medium at a density of 33.000 cells/ cm^2^ onto 0.1% gelatine coated wells in fibroblast medium 24 h before hiPS cell split. HiPS cells were cultured on a feeder cell layer in iPS medium containing DMEM/ F12, 20% knockout serum replacement, 1% Penicillin/ Streptomycin, 1% GlutaMAX, 1% MEM non essential amino acids, 0.2% 2-mercaptoethanol, and 10 to 15 ng/ ml hFGF-2 (Globalstem, Rockeville, USA). hiPS cells on matrigel (BD Biosciences, Heidelberg, Germany) were cultured in mTESR1 medium (Stemcell Technologies, Grenoble, France). Medium was changed daily and cells were passaged weekly using 10 μM ROCK inhibitor Y-27632 (Stemgent, Cambridge, USA) for increased plating efficiency. HiPS cells growing on a feeder cell layer were split mechanically weekly using pulled glass hooks by performing the cut and paste technique. Cells growing on matrigel were harvested enzymatically using 1 mg/ ml dispase (Stemcell Technologies, Grenoble, France) for 7 min and large bore tips to break down large clumps according to manufacturer’s recommendations. HEK293FT cells (Invitrogen, Darmstadt, Gemany) used to obtain the viral vectors were cultivated in fibroblast medium without Penicillin/ Streptomycin. All cells were cultivated at 37°C in a saturated humidity atmosphere containing 5% CO_2_.

### Generation of retroviruses

Retroviral pMIG vectors containing the cDNA of the human genes Oct4, Sox2, Klf4, and c-Myc were used as described recently [32]. Briefly, 3×10^6^ HEK293FT cells per 10 cm-dish were seeded onto ten dishes and incubated overnight. A solution containing 2.5 μg retroviral vector encoding for GFP and one of the transcription factors (Sox2, Klf4, Oct4, or c-Myc) was incubated with 0.25 μg VSV-G and 2.25 μg Gag-Pol in X-tremeGENE9 (Roche, Mannheim, Germany)/ DMEM High Glucose mixture (1:4) which was added to each of the dishes. Medium was renewed after 18 h and cells were incubated further for 48 h. Subsequently, the virus-containing medium was collected and passed through a 0.45 μm filter. To concentrate the virus, the medium was centrifuged at 70.000 × g at 4°C for 90 min, resuspended in 0.1 to 1 ml DMEM medium, and stored at −80°C. All four vectors contained a GFP sequence thus enabling titering by determining the percentage of GFP positive HEK293FT cells using FACS. Therefore, 1×10^5^ HEK293FT cells were seeded per 12-well in Penicillin/ Streptomycin free fibroblast medium containing 5 μg/ ml protamine sulfate and concentrated virus in the following volumes: 6.25 μl, 12.5 μl, 25 μl, and 50 μl. After 48 h cells were washed with PBS containing Ca^2+^/ Mg^2+^, trypsinized and centrifuged for 5 min at 500 × g. Pellet was resuspended in 100 μl PBS without Ca^2+^/ Mg^2+^ and fixed by adding 100 μl of 4% paraformaldehyde for 15 min. Afterwards, the percentage of GFP positive cells was determined via FACS analysis.

### Transduction of human fibroblasts

For transduction, 1×10^5^ fibroblasts were seeded per cavity of a 6-well plate and cultured for 18 h in fibroblast medium without Penicillin/ Streptomycin. Afterwards, fibroblast medium without Penicillin/ Streptomycin was supplemented with a volume of retrovirus of Sox2, Oct4, Klf4 (corresponding to 70 – 80% infection efficiency), and c-Myc (corresponding to 40–50% infection efficiency) in the presence of 5 μg/ ml protamine sulfate. Cells were incubated for 48 h. Subsequently, medium was aspirated and cells were washed twice with PBS containing Ca^2+^/ Mg^2+^. Transduced cells were trypsinized and reseeded onto a gelatin coated 6 cm-dish. The next day, medium was replaced with iPS medium supplemented with 0.5 mM valproic acid to further increase the efficiency of reprogramming. Medium was changed daily and valproic acid was omitted after seven days.

### Generation of hiPS cell lines

Initial hiPS colonies were routinely observed after three to four weeks. For further cultivation, they were picked using a 100 μl pipette tip and a pulled glass hook. Single colonies were transferred to the cavities of a 24-well plate, coated with 0.1% gelatin and 45.000 feeder cells/ cm^2^. After 4 to 7 days of proliferation hiPS colonies were mechanically divided into two to four pieces and further expanded. Within six weeks each single hiPS colony was expanded to obtain different clones.

### Karyotyping

Karyotyping was performed by Giemsa Trypsin banding. In short, colonies were incubated with a colcemid solution (10 μg/ ml in HBSS) for three hours to arrest cells in metaphase. Cells were treated with trypsin (0.25%) and the enzymatic reaction was stopped with Amniomax solution (Invitrogen, Darmstadt, Germany). Cells were centrifuged at 300 × g for 10 min and the pellet was resuspended in 4 ml hypotonic potassium chloride solution (5.62%). Cells were incubated for 5 min at 37°C and centrifuged at 300 × g for 10 min. The cells were resuspended and fixed in 5 ml glacial acetic acid and methanol (1:3) and subsequently centrifuged for 7 min at 350 × g. This step was repeated once. Finally, most of the supernatant was removed and cells were resuspended. Cell suspension was dropped onto cold slides and dried at 100°C for 1 h. Giemsa solution (5%) was added and incubated for 5 min. Slides were washed in distilled water two times, dried at room temperature and sealed with cover slips.

### Sequencing

Genomic DNA of fibroblasts, iPS cells grown on matrigel or neural progenitor cells were isolated using AllPrep Kit (Qiagen, Hilden, Germany) according to the manufacturer’s recommendations. Exon regions were amplified using HotStart Taq (Qiagen, Hilden, Germany) as follows: 95°C for 15 min followed by 13 cycles of 94°C for 30 s, 66°C for 30 s with 1.5°C decrease/ cycle and 72°C for 20 s; followed by 8 cycles of 94°C for 30 s, 46.5°C for 30 s with 1°C increase/ cycle and 72°C for 20 s; followed by 13 cycles of 94°C for 30 s, 66°C for 30 s with 1.5°C decrease/ cycle and 72°C for 20 s; followed by 11 cycles of 94°C for 30 s, 54.5°C for 30 s with 1°C increase/ cycle and 72°C for 20 s. Products were purified using ExoSAP Kit (USB Europe GmbH, Staufen, Germany) according to manufacturer’s recommendations. Sequence analysis was performed on a 3130XL Genetic Analyzer (Applied Biosystems, Carlsbad, USA).

### Alkaline phosphatase staining

HiPSCs were cultivated on a feeder cell layer for five days. Medium was removed, cells were washed with PBS and fixed with ice-cold methanol (100%) for 10 min at −20°C. Methanol was removed and cells were washed with PBS. Subsequently, cells were incubated at room temperature for 15 min with the staining solution: 75% distilled water, 10% sodium chloride solution (1 M), 10% Tris solution (1 M, pH 9.8), 5% magnesium chloride solution (1 M), and NBT/ BCIP solution (1:50, Roche, Mannheim, Germany). Staining solution was removed and cells were washed with distilled water. Microphotographs were taken using a Nikon Eclipse TS100 (Nikon, Düsseldorf, Germany).

### Immunocytochemistry

Cells were fixed at room temperature for 15 minutes in 4% paraformaldehyde, washed with PBS and stored in 0.02% NaN_3_ at 4°C. Immunocytochemistry was performed for Nanog (1:100, rabbit IgG polyclonal), Oct4 (1:100, rabbit IgG polyclonal), SSEA3 (1:100, rat IgM), SSEA4 (1:100, mouse IgG_3_), Tra-1-60 (1:100, mouse IgM), Tra-1-81 (1:100, mouse IgM, all Stemgent, Cambridge, USA), Smooth muscle actin (SMA, 1:50, mouse monoclonal, Dako, Glostrup, Denmark), alpha fetoprotein (alpha FP, 1:500, mouse monoclonal IgG, Sigma-Aldrich, Hamburg, Germany), Nestin (1:100, mouse monoclonal, R&D, Wiesbaden, Germany), MAP2ab (1:100, mouse monoclonal, Chemicon, Schwalbach, Gemany), Tuj1 (1:100, mouse monoclonal Tu-20, Santa Cruz biotechnology, Heidelberg, Germany) and Sox-2 (1:200, rabbit monoclonal, Abcam, Cambridge, UK). Blocking and permeabilization was carried out using 0.3% Triton X-100 and 5% normal goat serum (Dako, Glostrup, Denmark) for 30 min at room temperature. Cells were incubated with primary antibodies for 3 hours at room temperature in 1% normal goat serum, followed by three washing steps with PBS. Alexa Fluor 568 (1:1000, goat anti-mouse IgG or goat anti-rabbit IgG, Invitrogen, Darmstadt, Germany), Alexa Fluor 488 (1:1000, goat anti-mouse IgG or goat anti-rabbit IgG, Invitrogen, Darmstadt, Germany), or Alexa Fluor 488 (1:1000, goat anti-mouse IgM or goat anti-rat IgM, Invitrogen, Darmstadt, Germany) were used as secondary antibodies, incubated 1 h at room temperature with 1% normal goat serum in PBS. After washing with PBS, cells were stained with DAPI (5 minutes, 250 ng/ ml), washed three times and mounted with Mowiol-DABCO mounting medium. Pictures were taken with a Biozero 8000 microscope system (Keyence, Hamburg, Germany).

### Generation of embryoid bodies

To generate embryoid bodies (EBs), whole hiPS colonies were mechanically lifted off the feeder cell layer and transferred into a 15 ml conical tube. Once the colonies settled at the bottom of the conical tube, the medium was removed and 5 ml of differentiation medium, containing knockout DMEM, 20% FBS, 1% MEM non-essential amino acids, 2 mM GlutaMAX, and 0.1 mM beta-mercaptoethanol, was added. Afterwards, colonies were transferred into the cavity of a low attachment 6-well plate and incubated at 37°C/ 5% CO_2_. Medium was changed every second day until EBs were formed. After five to seven days EBs were transferred onto gelatin coated glass cover slips and supplied with differentiation medium. Once EBs were attached, medium was changed every second or third day. After 10 days of random differentiation, spread cells were washed with PBS and fixed with 4% PFA for 15 min. Fixed cells were washed with PBS and immunocytochemical stainings for nestin (ectoderm), smooth muscle actin (mesoderm), and alpha-fetoprotein (endoderm) were performed.

### Teratoma formation assay

Immunodeficient (SCID) hairless mice (Charles River Laboratories, Sulzfeld, Germany) were used for the teratoma formation assay. HiPSCs for injection were cultured on feeder cells in 6-well culture plates. For each injection the amount of 3 cavities of a 6-well culture plate were collected mechanically and centrifuged for 2 min at 200 × g. The pellet was resuspended in 1 ml of 0.25% trypsin/ EDTA. After 1 min, the reaction was stopped by adding 2 ml of fibroblast medium and centrifuged again for 2 min at 200 × g. Cells were resuspended in 140 μl of cold DMEM/ F12 and stored on ice. Directly before injection, cell suspension was mixed with 60 μl matrigel. Cells were injected subcutaneously into the flank of the hind limb. After 8–12 weeks, when tumors were clearly visible, the animals were sacrificed and tumors were removed. Tumor tissue was fixed in 4% formalin for 12 to 18 hours and embedded in paraffin for subsequent staining.

### H&E staining of tumor sections

4 μm thick tumor tissue sections were deparaffinized in xylol for 10 min and a descending ethanol concentration for 5 min each. Afterwards, the sections were washed in distilled water and stained with Mayers hematoxylin (Merck, Darmstadt, Germany) for 1 min. Next, the tissue was washed two times in distilled water and stained with eosin Y (Sigma-Aldrich, Hamburg, Germany) for 2 min. The slides were washed again twice and then dehydrated using an ascending ethanol concentrattion and xylol. Slides were mounted in Mowiol-DABCO. Microphotographs were taken with a Biozero 8000 microscope system (Keyence, Hamburg, Germany).

### Neural differentiation

To differentiate hiPS colonies into neural direction, the colonies were cut, transferred to Poly-L-ornithine (15 μg/ ml)/ laminin (10 μg/ ml) coated dishes, and cultivated for 10 days in medium consisting of Neurobasal, DMEM/ F12, 1xN2 supplement, 1xB27 supplement, GlutaMAX (2 mM) complemented with mouse recombinant noggin F_c_-chimera (500 ng/ ml, R&D, Wiesbaden, Germany), SB431542 (20 μM, Sigma-Aldrich, Hamburg, Germany) and hFGF-2 (5 ng/ ml, GlobalStem, Rockeville, USA). Neural rosettes were manually isolated using pulled glass hooks, gently trypsinized, and seeded as single cells on Poly-L-ornithine/ laminin coated dishes in medium consisting of Neurobasal, DMEM/ F12, 1×N2, 1×B27, and GlutaMAX (2 mM) supplemented with hFGF-2 (10 ng/ ml) and hEGF (10 ng/ ml, Peprotech, Hamburg, Germany). Neural progenitor cells were seeded at high densities (100–150.000 cells/ cm^2^) and passaged one day after reaching confluence using Trypsin/ Benzonase. Differentiation was induced by seeding the cells at a density of 50.000 cells/ cm^2^ and withdrawal of growth factors in the presence of BDNF (20 ng/ ml, Peprotech, Hamburg, Germany).

### Patch clamp recordings

Patch clamp recordings were performed using an EPC-10 amplifier (Heka, Lambrecht, Germany). Patch pipettes were pulled from borosilicate glass tubing (Harvard Apparatus, Holliston, USA). The internal solution contained (mM): KCl 130, NaCl 10, HEPES 10, EGTA 11, MgCl_2_×6H_2_O 1, CaCl_2_×H_2_O 2, Mg-ATP 2. pH was adjusted to 7.2. When filled, electrodes had a resistance of 6–8 MΩ. Cell cultures were continuously superfused with an extracellular solution, consisting of (mM): NaCl 125, KCl 2.5, CaCl_2_×H_2_O 2, MgCl_2_×6H_2_O 1, NaHCO_3_ 26, NaH_2_PO_4_×H_2_O 1.25, glucose×H_2_O 25. Solution was continuously bubbled with carbogen to maintain a pH of 7.4. Recordings were made in the whole cell configuration with holding potentials (VH) of −60 or −80 mV. Current voltage responses were evoked by applying 100 ms voltage steps from −60 mV to +50 mV in 10 mV increments. Data were filtered at 3 kHz, digitized and stored on-line using Pulse (Heka, Lambrecht, Germany). Na^+^ and K^+^ currents were identified via their I-V relationship. Na^+^ currents were antagonized in some experiments by TTX (1 μM). Current clamp mode was used to apply current steps to induce action potentials or to measure spontaneous action potentials. Postsynaptic currents were measured in the voltage clamp mode at a VH of −60 mV. Mini Analysis 6 (Synaptosoft, USA) was used to analyse recordings of post-synaptic currents. Data are given as mean ± SEM.

### Filipin staining

Filipin is a polyene antibiotic which binds to free cholesterol and is widely used to analyze the sequestration of unesterified cholesterol in NPC1-deficient cells. Therefore, cells were fixed with 4% paraformaldehyde (in PBS) for 15 min, washed with PBS and incubated at room temperature for 45 min in the dark with a staining solution containing 100 μg/ ml Filipin (Polysciences, Eppelheim, Germany) in PBS. Cells were washed twice with PBS for 5 min. Slides were mounted using Mowiol-DABCO and sealed with cover slips. After 12 h of drying in the dark at room temperature, fluorescence pictures were taken with a Biozero 8000 microscope (Keyence, Hamburg, Germany).

### Amplex red assay

To quantify the amount of cholesterol we used the Amplex Red cholesterol assay [33]. Fibroblasts, iPS cells grown on matrigel and neural progenitor cells were harvested in PBS/ SDS (0.1%) at room temperature, sheared through a 27 G needle and cholesterol levels were determined using the Amplex Red cholesterol assay kit (Molecular Probes, Darmstadt, Gemany) according to the manufacturer’s instructions. Protein concentrations in lysates were measured using the bicinchoninic acid assay (BCA, Pierce, USA).

### Statistical analysis

Analysis of the data was carried out with GraphPad Prism 5 (GraphPad Software Inc., USA). Data are given as mean ± SEM. Unless otherwise stated, unpaired t-tests were used to test for significance, with * = p<0.05 and ** = p<0.01, *** = p<0.001.

## Results

### Reprogramming of mutNPC1 and wtNPC1 fibroblasts

We reprogrammed fibroblasts originating from a male patient with an early-infantile form of NPC1 characterized by massive accumulation of unesterified cholesterol in lysosomal and late endosomal structures. Cells derived from this donor will be referred to as mutNPC1 and cells derived from age- and sex-matched fibroblasts of a healthy individual will be referred to as wtNPC1.

**Figure 1:**
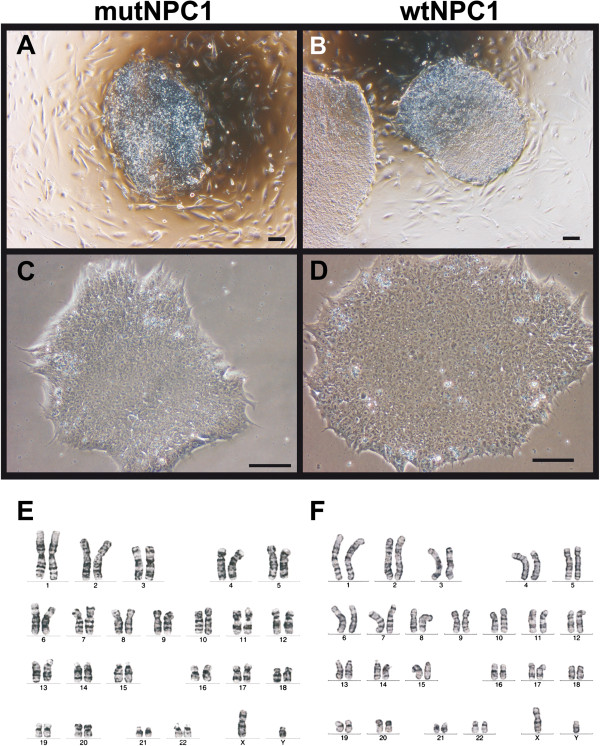
**Colonies of fibroblast-derived human iPSCs.** Images of hiPSC colonies cultured on mouse feeder cells **(A,B)** or matrigel **(C,D)**. The colonies could be easily distinguished by their morphology, showing a high nuclear to cytoplasm ratio and sharp borders, resembling the morphology of human embryonic stem cells (scale bars = 100 μm). The hiPSCs-derived from fibroblasts of a NPC1-patient **(E)** and unaffected individual **(F)** displayed a normal karyotype. Karyotyping was performed by Giemsa Trypsin banding.

After three to four weeks of cultivation, the first hiPSC colonies appeared characterized by their embryonic stem (ES) cell-like morphology, e.g. round to oval shape with a sharp border and a high nuclear to cytoplasm ratio (Figure 1A-D). Mechanically isolated colonies were expanded to hiPSC lines on irradiated mouse embryonic fibroblasts and later also on matrigel (Figure 1C,D). The morphology of mutNPC1 and wtNPC1 hiPSCs was similar in both culture systems. The karyotype of the cells was analyzed to rule out any chromosomal abnormalities, which may have arisen during reprogramming, where our hiPSCs displayed a normal karyotype (Figure 1E,F). Sequencing of the hiPSCs revealed that the mutations in the NPC1 gene were maintained (data not shown).

### Pluripotency of mutNPC1 and wtNPC1 hiPSCs

**Figure 2:**
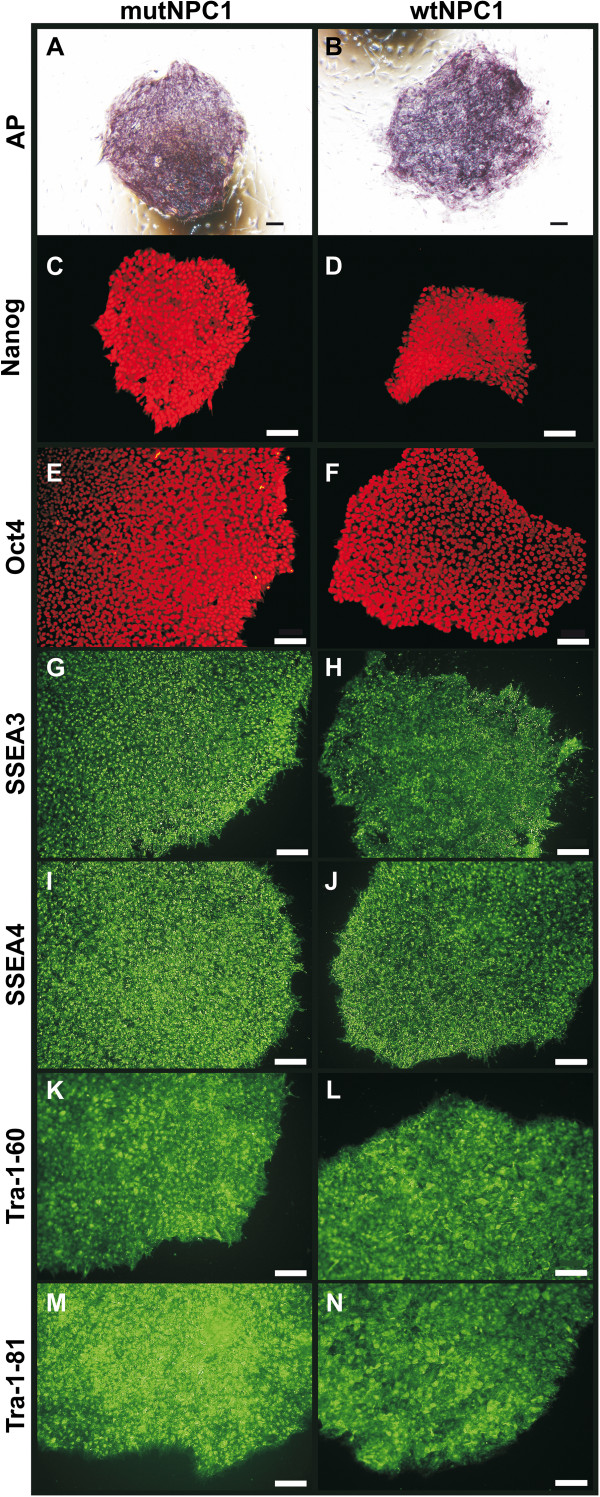
**Detection of pluripotency markers in hiPSCs.** hiPSCs-derived from fibroblasts of NPC1 patient (mutNPC1) and unaffected control (wtNPC1) showed a strong alkaline phosphatase (AP) staining **(A,B)** and expressed a set of pluripotency markers **(C-N)** demonstrating the pluripotent state of the cells (scale bars = 100 μm).

**Figure 3:**
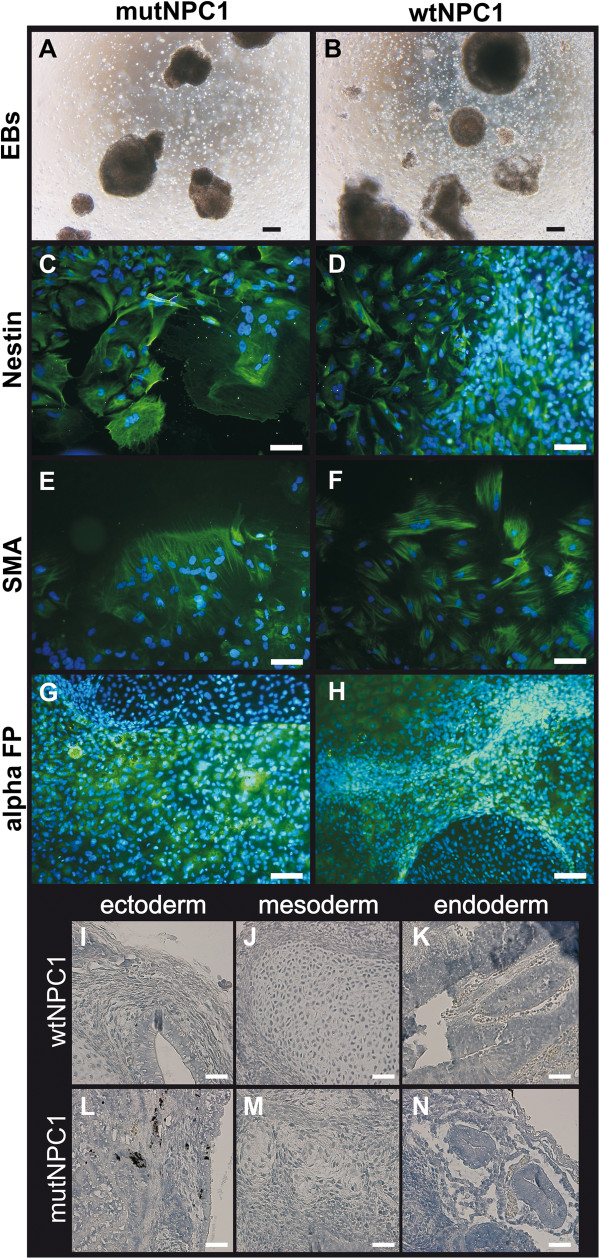
**Embryoid body formation and teratoma induction.** Free floating embryoid bodies (EBs) of mutNPC1 and wtNPC1 hiPSCs were generated **(A,B)**, which subsequently spontaneously differentiated **(C-H)**. EBs of both cell lines were positive for markers of the three germ layers, namely Nestin **(C,D)** for ectoderm, smooth muscle actin (SMA) for mesoderm **(E,F)**, and alpha fetoprotein (alpha FP) for endoderm **(G,H)**. Nuclei were stained by DAPI, shown in blue, staining of Nestin, SMA and alpha FP is shown in green (scale bars **A-H** = 100 μm). HiPSCs of the mutNPC1 and wtNPC1 cell lines were injected in immunodeficient mice and tumors were extracted after 8–12 weeks. MutNPC1 and wtNPC1 hiPSCs induced teratomas contained structures typical for the ectoderm **(I,J)**, mesoderm **(K,L)**, and endoderm (**M,N**, scale bars I-N = 50 μm).

HiPSCs derived from of mutNPC1 and wtNPC1 human fibroblasts were characterized regarding their pluripotency. First, we analyzed the alkaline phosphatase (AP) expression. All hiPSCs colonies demonstrated strong AP expression (Figure 2A,B). The expression of several transcription factors and surface markers was determined by immunocytochemistry. HiPSCs displayed a high expression of the transcription factors Nanog (Figure 2C,D) and Oct4 (Figure 2E,F). The glycosphingolipids SSEA3 and SSEA4 (Figure 2G-J), were strongly expressed as well as the keratan sulfate antigens Tra-1-60 and Tra-1-81 (Figure 2K-N). No obvious differences between mutNPC1 and wtNPC1 cells in marker expression could be observed. The spontaneous differentiation by embryoid body (EB) formation into cells of all three germ layers was also used to verify the pluripotency (Figure 3A-H). Herein, cells from all three germ layers were identified, thus proving the pluripotency of the hiPSCs *in vitro*. The induction of teratoma was used as an *in vivo* pluripotency assay. The hiPSCs induced teratomas in immunodeficent mice, and the analysis of the tumors revealed tissues of all three germ layers (Figure 3I-N).

### Neuronal differentiation of mutNPC1 and wtNPC1 hiPS cells

**Figure 4:**
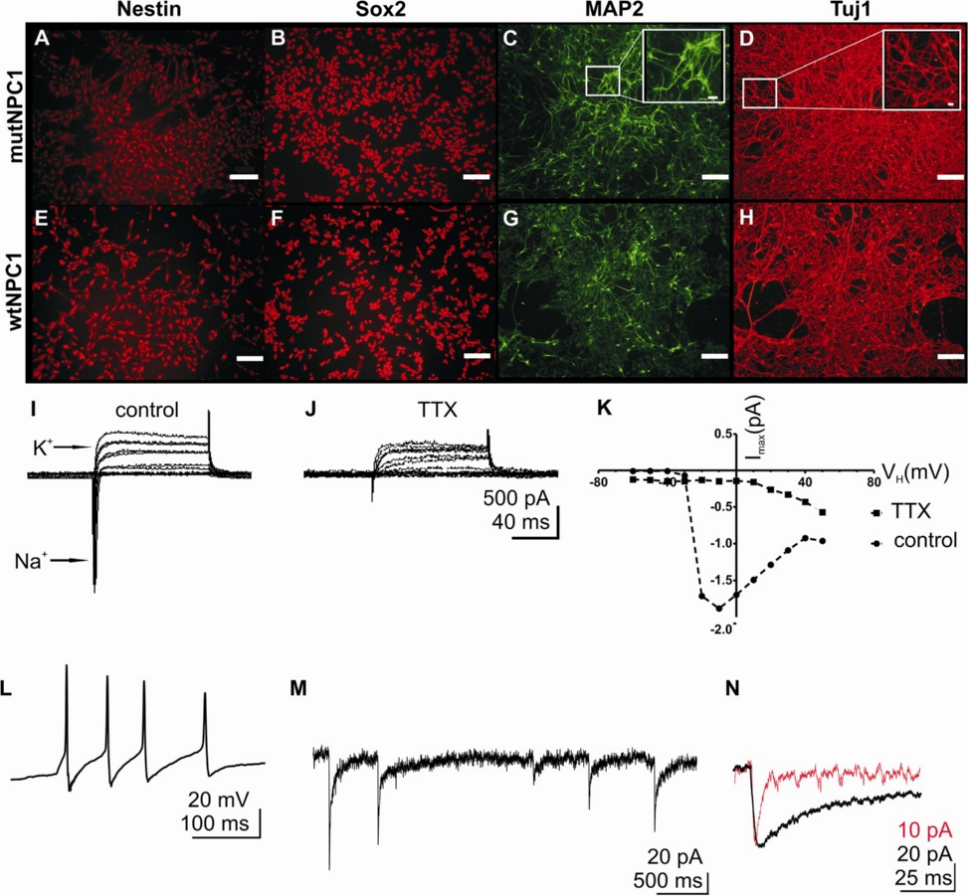
**Differentiation of neural progenitor cells.** HiPSC cell lines were directed to neural progenitor cells **(A,B,E,F)** and subsequently differentiated into neuronal cells. Neural progenitor cells expressed typical markers like Nestin (**A,E,** shown in red) or Sox2 (**B,F** shown in red). Differentiation of neural progenitor cells was induced by withdrawal of growth factors and resulted in cells demonstrating a neuronal morphology, expressing neuronal markers like MAP2 (**C,G** shown in green) and Tuj1 (**D,H** shown in red). Inset in **C** and **D** shows an example of a higher magnification to demonstrate the dense network of processes built up by the cells during differentiation. Patch clamp experiments were performed to analyze the maturation of the cells into functional neuronal cells. Voltage steps elicited inward and outward directed Na^+^ and K^+^ currents **(I)**, where Na^+^ currents could be blocked by TTX **(J)** and possessed I/ V relationship typical for voltage gated Na^+^ channels **(K)**. The example shows recordings of a mutNPC1 cell, differentiated for four weeks. After 7–9 weeks of differentiation, we observed spontaneous action potentials **(L)** as well as spontaneous postsynaptic currents **(M)**. **(N)** shows a superimposed average of spontaneous postsynaptic currents with fast decay kinetics (red trace) and slow decay kinetics (black trace), indicating input of different synaptic sites. The example shows recordings of a mutNPC1 cell, differentiated for 7 weeks.

In a last step, we generated neural progenitor cells, which were positive for Nestin and Sox2 (Figure 4A,B,E,F). Differentiated neural progenitor cells expressed neuronal markers like MAP2 (Figure 4C,G), and Tuj1 (Figure 4D,H) demonstrating the neuronal phenotype of the cells. Furthermore, we proved the differentiation into functional neuronal cells by means of patch clamp recordings. In these experiments we observed voltage dependent Na^+^ and K^+^ channels (Na_V_s and K_V_s) (Figure 4I) after three to four weeks of differentiation, where inward currents could be blocked by TTX (Figure 4K). Although the cells exhibited Na_V_s, they did not demonstrate any spontaneous action potentials in the current clamp mode. But, we observed spontaneous action potentials after 7–8 weeks of differentiation (Figure 4L). In addition, we recorded spontaneous postsynaptic currents. An example of a mutNPC1 cell is shown in Figure 4M. The analysis of the decay kinetics (Figure 4N) revealed a rise time of 2.1 ± 1.1 ms. Analysing the decay kinetics we found a group of post synaptic currents best fitted by a mono-exponential function (τ: 3.5 ± 0.4 ms, Figure 4N, black trace) with a mean amplitude of 29.5 ± 1.1 pA, and a group best fitted by a bi-exponential function (τ1: 9.9 ± 1.9 ms, τ2: 124.7 ± 23.8 ms, Figure 4N, red trace) with a mean amplitude of 68.1 ± 7.1 pA.

### mutNPC1 cells accumulated cholesterol

**Figure 5:**
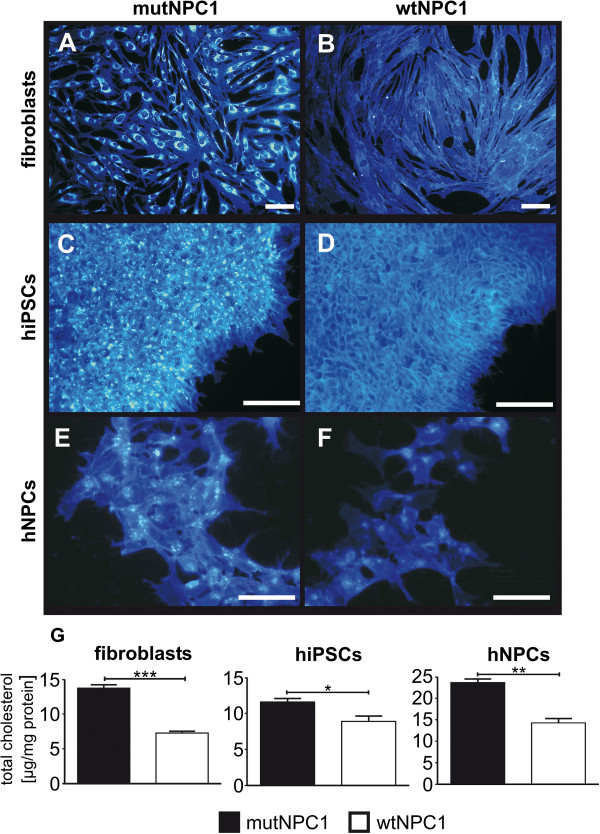
**Cholesterol accumulation in fibroblasts, hiPSCs, and neural progenitor cells.** Cholesterol accumulation is one of the hallmarks of NPC1 disease. Filipin stainings of fibroblast (**A,B**, shown in blue) are used for diagnostics, where fibroblasts of NPC1 patients with a “classic” biochemical phenotype demonstrate a clear perinuclear accumulation **(A)** in contrast to fibroblasts of an unaffected individual **(B)**. These differences were found in hiPSCs **(C,D)** and neural progenitor cells (hNPCs, **E,F**) derived from mutNPC1 and wtNPC1 fibroblasts. (scale bar = 100 μm). A quantification of the amount of cholesterol **(G)** in fibroblasts, iPSCs, and hNPCs revealed elevated cholesterol levels in mutNPC1 cell lines (black bars) in contrast to wtNPC1 cell lines (white bars). The total amount differed slightly between the cell lines but the relative proportion was comparable.

The hallmark of NPC1 is abnormal cholesterol trafficking resulting in an accumulation of cholesterol. Free cholesterol can be visualized by Filipin. An analysis of the cholesterol distribution in mutNPC1 fibroblasts, iPSCs, and derived neural progenitor cells (Figure 5A,C,E) revealed an accumulation of cholesterol. In contrast, an accumulation was not detectable in the fibroblasts, iPSCs, or neural progenitor cells of the wtNPC1 counterpart (Figure 5B,D,F). As a next step, we used the Amplex Red assay [33] to confirm and quantify the observed cholesterol accumulations. The experiments revealed a significantly increased cholesterol content in mutNPC1 cells in comparison to wtNPC1 cells (Figure 5G) (fibroblasts: mutNPC1: 13.7 ± 0.5 μg vs. wtNPC1: 7.3 ± 0.3 μg; iPSCs: mutNPC1: 11.6 ± 0.6 μg vs. wtNPC1: 8.9 ± 0.7 μg; neural progenitor cells: mutNPC1: 23.6 ± 0.9 μg vs. wtNPC1: 14.2 ± 1.6 μg).

## Discussion

In this study we aimed to reprogram fibroblasts originating from a NPC1 patient with an early-infantile form of the disease. Therefore, we used retroviruses expressing Oct4, Klf4, Sox2, and c-Myc in combination with GFP. These factors have been described previously to be efficient in generating hiPSCs [34]. The retroviral particles used in this study were successfully used to reprogram skin fibroblasts of Parkinsons disease into hiPSCs [32]. HiPS colonies were chosen based on an absent GFP-signal indicating a silenced expression of transcription factors [35] and were subcultured to stable hiPSC lines. The obtained mutNPC1 and wtNPC1 hiPSC lines were characterized by their ES-cell like morphology, the expression of alkaline phosphatase, and the pluripotency markers Nanog, Oct4, SSEA3, SSEA4, Tra-1-60, Tra-1-81. A comparable expression of Tra-1-81 was reported for NPC1 knock-down and control ES cells [36]. However, comparative analyses of additional pluripotency markers were not performed in this study. We found no obvious differences between mutNPC1 and wtNPC1 cells in pluripotency marker expression. These results are in accordance with other studies dealing with patient-specific induced pluripotent stem cells in a variety of other diseases [9]. However, to our knowledge this is the first study describing the expression of pluripotency markers SSEA3, SSEA4, and Tra-1-60 in pluripotent cells harboring disease-causing mutations in the NPC1 gene. In addition, the widely known risk of chromosomal abnormalities, potentially occurring during iPS generation and expansion [37], did not arise in our cell lines as proved by karyotyping. The spontaneous differentiation by embryoid body (EB) formation [38] into cells of all three germ layers and the induction of teratomas in immunodeficient mice [39] further demonstrated the pluripotent state of the mutNPC1 and wtNPC1 hiPSCs.

We further differentiated the hiPSCs into neural progenitor cells to generate a suitable *in vitro* disease model. So far, two human cellular neural models based on NPC1-knockdown have been reported. These include SH-SY5Y neuroblastoma cells [40] and human embryonic stem cells [36], which resemble the phenotype only in some aspects of the NPC1 disease. Therefore, they are not an appropriate model to analyze the influence of specific mutations in a patient-specific (epi)genetic background. Here, we generated homogenous neural progenitor cells based on mutNPC1 and wtNPC1 hiPSCs, which were positive for neural markers Nestin and Sox2 [41]. In contrast, Ordonez et al. [36] obtained a homogenous population of neural stem cells from control hESCs but not from NPC1 knock-down hESCs. The authors speculate that these findings might be based on the genetic background of the cells [36].

Our differentiated neural progenitor cells expressed the neuronal markers MAP2 and Tuj1. We did not observe obvious morphological differences between mutNPC1 and wtNPC1 neuronal cells. In contrast, distortion of neuronal shape and extensive growth of ectopic neurites have been reported for multipotent adult stem cells (MASCs) derived cells [30]. However, a further detailed analysis of our neural progenitor cells and derived neuronal cells will be performed to analyze changed morphology of neuronal cells and perturbances of proliferation, described for murine neural stem cells [42]. In a first set of experiments, we demonstrated the differentiation of neural progenitor cells into functional neuronal cells by means of patch clamp recordings. We observed voltage dependent Na^+^ and K^+^ channels, where inward currents were blocked by TTX. Spontaneous action potentials and postsynaptic currents could only be observed in cells differentiated for 7–8 weeks, indicating the maturation time of human neural progenitor cells to functional neurons, as described for interneurons derived from hiPSCs [43,44]. The here recorded spontaneous postsynaptic currents displayed different amplitudes and the time constants of the current decay could be fitted with mono-exponential or bi-exponential functions. The differences between the time constants may indicate different types of synaptic input, where currents with small amplitudes and fast mono-exponential decay suggest excitatory and events with larger amplitudes and slow bi-exponential decay suggest inhibitory input [45,46]. These preliminary results indicate that the differentiated cells are able to build up chemical synapses. This is of special interest as recent studies described disturbed transmitter release in NPC1 deficient mice, where a higher rate of glutamate release was observed leading to higher frequency of excitatory postsynaptic currents [47]. Thus, our cells provide a platform to study such alterations in synaptic transmission in human neuronal cells gained from different individuals. Ultimately, these results demonstrate a maturation into functional neuronal cells, where future studies will focus on the nature of the expressed voltage and ligand gated ion channels in mutNPC1 and wtNPC1 neuronal cells.

Our neural progenitor cells were analyzed regarding their impaired cholesterol trafficking by Filipin. It visualizes free cholesterol and is routinely used for human dermal fibroblasts in the diagnostics of the NPC1 disease [20]. We found clear cholesterol accumulation in mutNPC1 fibroblasts, hiPSCs, and derived neural progenitor cells. In contrast, such an accumulation was not observed in the fibroblasts, hiPSCs, or neural progenitor cells of the wtNPC1 counterpart. The accumulation pattern of cholesterol in the herein described cells was comparable to accumulations described in a NPC1 knock-down mouse model [48], and SH-SY5Y neuroblastoma cells [40]. Recently, a neural model based on multipotent adult stem cells was described [30]. The neural differentiated progeny of these cells, demonstrated a comparable accumulation of cholesterol, where this derivation method is only applicable to early passages (<3 passages) of fibroblasts, potentially limiting its use with characterized cell lines from cell repositories.

Finally, we used the Amplex Red assay to confirm and quantify the observed cholesterol accumulations in our cells. These experiments revealed significantly increased cholesterol content in mutNPC1 cells in comparison to wtNPC1 cells, which was conserved in fibroblasts, hiPSCs, and derived neural progenitor cells. To our knowledge this is the first analysis of cholesterol distribution using Filipin staining and cholesterol quantification in hiPSCs and neural progenitor cells derived from human NPC1 deficient fibroblasts.

## Conclusion

In this study we generated, for the first time, induced pluripotent stem cells derived from fibroblasts of a NPC1 patient. The cells demonstrated an accumulation of cholesterol, resembling the phenotype of NPC1 deficient cells, and can provide an *in vitro* model of NPC1. We are convinced that the here reported hiPSCs and the derived neural progenitor cells are an excellent model to study the influence of the specific mutation on the phenotype, e.g. consequences of a misfolded NPC1 protein. Moreover, the cells provide the opportunity to analyze the consequences of a NPC1 mutation on the patient-specific (epi)genetic background, and will thus serve to elucidate further the pathogenic mechanisms of this fatal lysosomal storage disorder.

## Competing interests

The authors declare that they have no competing interests.

## Authors’ contributions

MT: conception and design, collection and/ or assembly of data, data analysis and interpretation, manuscript writing. RH: conception and design, collection and/ or assembly of data, data analysis and interpretation, manuscript writing. PS: collection and/ or assembly of data, manuscript writing. CK: collection and/ or assembly of data, manuscript writing. AR: conception and design, manuscript writing, final approval of manuscript. MJF: conception and design, collection and/ or assembly of data, data analysis and interpretation, manuscript writing, final approval of manuscript. All authors read and approved the final manuscript.

## References

[B1] Takahashi K Tanabe K Ohnuki M Narita M Ichisaka T Tomoda K Yamanaka S Induction of pluripotent stem cells from adult human fibroblasts by defined factors Cell 2007 131 861 872 10.1016/j.cell.2007.11.019 18035408

[B2] Park IH Zhao R West JA Yabuuchi A Huo H Ince TA Lerou PH Lensch MW Daley GQ Reprogramming of human somatic cells to pluripotency with defined factors Nature 2008 451 141 146 10.1038/nature06534 18157115

[B3] Ye Z Zhan H Mali P Dowey S Williams DM Jang YY Dang CV Spivak JL Moliterno AR Cheng L Human-induced pluripotent stem cells from blood cells of healthy donors and patients with acquired blood disorders Blood 2009 114 5473 5480 10.1182/blood-2009-04-217406 19797525 PMC2798863

[B4] Liu H Ye Z Kim Y Sharkis S Jang YY Generation of endoderm-derived human induced pluripotent stem cells from primary hepatocytes Hepatology 2010 51 1810 1819 10.1002/hep.23626 20432258 PMC2925460

[B5] Karumbayaram S Novitch BG Patterson M Umbach JA Richter L Lindgren A Conway AE Clark AT Goldman SA Plath K Directed differentiation of human-induced pluripotent stem cells generates active motor neurons Stem Cells 2009 27 806 811 10.1002/stem.31 19350680 PMC2895909

[B6] Zhang J Wilson GF Soerens AG Koonce CH Yu J Palecek SP Thomson JA Kamp TJ Functional cardiomyocytes derived from human induced pluripotent stem cells Circ Res 2009 104 e30 e41 10.1161/CIRCRESAHA.108.192237 19213953 PMC2741334

[B7] Zhang D Jiang W Liu M Sui X Yin X Chen S Shi Y Deng H Highly efficient differentiation of human ES cells and iPS cells into mature pancreatic insulin-producing cells Cell Res 2009 19 429 438 10.1038/cr.2009.28 19255591

[B8] Yang S Bo J Hu H Guo X Tian R Sun C Zhu Y Li P Liu P Zou S Derivation of male germ cells from induced pluripotent stem cells in vitro and in reconstituted seminiferous tubules Cell Prolif 2012 45 91 100 10.1111/j.1365-2184.2012.00811.x 22324506 PMC6496715

[B9] Park IH Arora N Huo H Maherali N Ahfeldt T Shimamura A Lensch MW Cowan C Hochedlinger K Daley GQ Disease-specific induced pluripotent stem cells Cell 2008 134 877 886 10.1016/j.cell.2008.07.041 18691744 PMC2633781

[B10] Rashid ST Corbineau S Hannan N Marciniak SJ Miranda E Alexander G Huang-Doran I Griffin J Ahrlund-Richter L Skepper J Modeling inherited metabolic disorders of the liver using human induced pluripotent stem cells J Clin Invest 2010 120 3127 3136 10.1172/JCI43122 20739751 PMC2929734

[B11] Jin ZB Okamoto S Osakada F Homma K Assawachananont J Hirami Y Iwata T Takahashi M Modeling retinal degeneration using patient-specific induced pluripotent stem cells PLoS ONE 2011 6 e17084 10.1371/journal.pone.0017084 21347327 PMC3037398

[B12] Zhang N An MC Montoro D Ellerby LM Characterization of human Huntington’s disease cell model from induced pluripotent stem cells PLoS Curr 2010 2 RRN1193 10.1371/currents.RRN1193 PMC2966296 21037797

[B13] Lemonnier T Blanchard S Toli D Roy E Bigou S Froissart R Rouvet I Vitry S Heard JM Bohl D Modeling neuronal defects associated with a lysosomal disorder using patient-derived induced pluripotent stem cells Hum Mol Genet 2011 20 3653 3666 10.1093/hmg/ddr285 21685203

[B14] Yokoo N Baba S Kaichi S Niwa A Mima T Doi H Yamanaka S Nakahata T Heike T The effects of cardioactive drugs on cardiomyocytes derived from human induced pluripotent stem cells Biochem Biophys Res Commun 2009 387 482 488 10.1016/j.bbrc.2009.07.052 19615974

[B15] Morris JA Zhang D Coleman KG Nagle J Pentchev PG Carstea ED The genomic organization and polymorphism analysis of the human Niemann-Pick C1 gene Biochem Biophys Res Commun 1999 261 493 498 10.1006/bbrc.1999.1070 10425213

[B16] Carstea ED Morris JA Coleman KG Loftus SK Zhang D Cummings C Gu J Rosenfeld MA Pavan WJ Krizman DB Niemann-Pick C1 disease gene: homology to mediators of cholesterol homeostasis Science 1997 277 228 231 10.1126/science.277.5323.228 9211849

[B17] Davies JP Ioannou YA Topological analysis of Niemann-Pick C1 protein reveals that the membrane orientation of the putative sterol-sensing domain is identical to those of 3-hydroxy-3-methylglutaryl-CoA reductase and sterol regulatory element binding protein cleavage-activating protein J Biol Chem 2000 275 24367 24374 10.1074/jbc.M002184200 10821832

[B18] Vanier MT Niemann-Pick disease type C Orphanet J Rare Dis 2010 5 16 10.1186/1750-1172-5-16 20525256 PMC2902432

[B19] Sokol J Blanchette-Mackie J Kruth HS Dwyer NK Amende LM Butler JD Robinson E Patel S Brady RO Comly ME Type C Niemann-Pick disease: lysosomal accumulation and defective intracellular mobilization of low density lipoprotein cholesterol J Biol Chem 1988 263 3411 3417 3277970

[B20] Wraith JE Baumgartner MR Bembi B Covanis A Levade T Mengel E Pineda M Sedel F Topcu M Vanier MT Recommendations on the diagnosis and management of Niemann-Pick disease type C Mol Genet Metab 2009 98 152 165 10.1016/j.ymgme.2009.06.008 19647672

[B21] Liscum L Ruggiero RM Faust JR The intracellular transport of low density lipoprotein-derived cholesterol is defective in Niemann-Pick type C fibroblasts J Cell Biol 1989 108 1625 1636 10.1083/jcb.108.5.1625 2715172 PMC2115531

[B22] Zampieri S Mellon SH Butters TD Nevyjel M Covey DF Bembi B Dardis A Oxidative stress in NPC1 deficient cells: protective effect of allopregnanolone J Cell Mol Med 2009 13 3786 3796 10.1111/j.1582-4934.2008.00493.x 18774957 PMC2832077

[B23] Kwon HJ Abi-Mosleh L Wang ML Deisenhofer J Goldstein JL Brown MS Infante RE Structure of N-terminal domain of NPC1 reveals distinct subdomains for binding and transfer of cholesterol Cell 2009 137 1213 1224 10.1016/j.cell.2009.03.049 19563754 PMC2739658

[B24] Loftus SK Morris JA Carstea ED Gu JZ Cummings C Brown A Ellison J Ohno K Rosenfeld MA Tagle DA Murine model of Niemann-Pick C disease: mutation in a cholesterol homeostasis gene Science 1997 277 232 235 10.1126/science.277.5323.232 9211850

[B25] Vite CH Ding W Bryan C O’Donnell P Cullen K Aleman D Haskins ME Van Winkle T Clinical, electrophysiological, and serum biochemical measures of progressive neurological and hepatic dysfunction in feline Niemann-Pick type C disease Pediatr Res 2008 64 544 549 10.1203/PDR.0b013e318184d2ce 18614965 PMC3251164

[B26] Huang X Suyama K Buchanan J Zhu AJ Scott MP A drosophila model of the Niemann-Pick type C lysosome storage disease: dnpc1a is required for molting and sterol homeostasis Development 2005 132 5115 5124 10.1242/dev.02079 16221727

[B27] Karten B Peake KB Vance JE Mechanisms and consequences of impaired lipid trafficking in Niemann-Pick type C1-deficient mammalian cells Biochim Biophys Acta 2009 1791 659 670 10.1016/j.bbalip.2009.01.025 19416638

[B28] Pentchev PG Gal AE Booth AD Omodeo-Sale F Fouks J Neumeyer BA Quirk JM Dawson G Brady RO A lysosomal storage disorder in mice characterized by a dual deficiency of sphingomyelinase and glucocerebrosidase Biochim Biophys Acta 1980 619 669 679 10.1016/0005-2760(80)90116-2 6257302

[B29] Walkley SU Suzuki K Consequences of NPC1 and NPC2 loss of function in mammalian neurons Biochim Biophys Acta 2004 1685 48 62 10.1016/j.bbalip.2004.08.011 15465426

[B30] Bergamin N Dardis A Beltrami A Cesselli D Rigo S Zampieri S Domenis R Bembi B Beltrami CA A human neuronal model of Niemann Pick C disease developed from stem cells isolated from patient’s skin Orphanet J Rare Dis 2013 8 34 10.1186/1750-1172-8-34 23433359 PMC3648447

[B31] Sun X Marks DL Park WD Wheatley CL Puri V O’Brien JF Kraft DL Lundquist PA Patterson MC Pagano RE Niemann-Pick C variant detection by altered sphingolipid trafficking and correlation with mutations within a specific domain of NPC1 Am J Hum Genet 2001 68 1361 1372 10.1086/320599 11349231 PMC1226123

[B32] Seibler P Graziotto J Jeong H Simunovic F Klein C Krainc D Mitochondrial parkin recruitment is impaired in neurons derived from mutant PINK1 induced pluripotent stem cells J Neurosci 2011 31 5970 5976 10.1523/JNEUROSCI.4441-10.2011 21508222 PMC3091622

[B33] Tängemo C Weber D Theiss S Mengel E Runz H Niemann-Pick type C disease: characterizing lipid levels in patients with variant lysosomal cholesterol storage J Lipid Res 2011 52 813 825 10.1194/jlr.P013524 21245028 PMC3284170

[B34] Huangfu D Maehr R Guo W Eijkelenboom A Snitow M Chen AE Melton DA Induction of pluripotent stem cells by defined factors is greatly improved by small-molecule compounds Nat Biotechnol 2008 26 795 797 10.1038/nbt1418 18568017 PMC6334647

[B35] Yao S Sukonnik T Kean T Bharadwaj RR Pasceri P Ellis J Retrovirus silencing, variegation, extinction, and memory are controlled by a dynamic interplay of multiple epigenetic modifications Mol Ther 2004 10 27 36 10.1016/j.ymthe.2004.04.007 15233939

[B36] Ordonez MP Roberts EA Kidwell CU Yuan SH Plaisted WC Goldstein LS Disruption and therapeutic rescue of autophagy in a human neuronal model of Niemann Pick type C1 Hum Mol Genet 2012 21 2651 2662 10.1093/hmg/dds090 22437840 PMC3363339

[B37] Mayshar Y Ben David U Lavon N Biancotti JC Yakir B Clark AT Plath K Lowry WE Benvenisty N Identification and classification of chromosomal aberrations in human induced pluripotent stem cells Cell Stem Cell 2010 7 521 531 10.1016/j.stem.2010.07.017 20887957

[B38] Sheridan SD Surampudi V Rao RR Analysis of embryoid bodies derived from human induced pluripotent stem cells as a means to assess pluripotency Stem Cells Int 2012 2012 738910 22550517 10.1155/2012/738910 PMC3328185

[B39] Lensch MW Schlaeger TM Zon LI Daley GQ Teratoma formation assays with human embryonic stem cells: a rationale for one type of human-animal chimera Cell Stem Cell 2007 1 253 258 10.1016/j.stem.2007.07.019 18371359

[B40] Rodriguez-Pascau L Coll MJ Casas J Vilageliu L Grinberg D Generation of a human neuronal stable cell model for Niemann-Pick C disease by RNA interference JIMD Rep 2012 4 29 37 23430894 10.1007/8904_2011_64 PMC3509875

[B41] Elkabetz Y Panagiotakos G Al Shamy G Socci ND Tabar V Studer L Human ES cell-derived neural rosettes reveal a functionally distinct early neural stem cell stage Genes Dev 2008 22 152 165 10.1101/gad.1616208 18198334 PMC2192751

[B42] Yang SR Kim SJ Byun KH Hutchinson B Lee BH Michikawa M Lee YS Kang KS NPC1 gene deficiency leads to lack of neural stem cell self-renewal and abnormal differentiation through activation of p38 mitogen-activated protein kinase signaling Stem Cells 2006 24 292 298 10.1634/stemcells.2005-0221 16099992

[B43] Nicholas CR Chen J Tang Y Southwell DG Chalmers N Vogt D Arnold CM Chen YJ Stanley EG Elefanty AG Functional maturation of hPSC-derived forebrain interneurons requires an extended timeline and mimics human neural development Cell Stem Cell 2013 12 573 586 10.1016/j.stem.2013.04.005 23642366 PMC3699205

[B44] Marin O Human cortical interneurons take their time Cell Stem Cell 2013 12 497 499 10.1016/j.stem.2013.04.017 23642355

[B45] Protti DA Gerschenfeld HM Llano I GABAergic and glycinergic IPSCs in ganglion cells of rat retinal slices J Neurosci 1997 17 6075 6085 9236219 10.1523/JNEUROSCI.17-16-06075.1997 PMC6568339

[B46] Frech MJ Perez-Leon J Wassle H Backus KH Characterization of the spontaneous synaptic activity of amacrine cells in the mouse retina J Neurophysiol 2001 86 1632 1643 11600626 10.1152/jn.2001.86.4.1632

[B47] Wasser CR Ertunc M Liu X Kavalali ET Cholesterol-dependent balance between evoked and spontaneous synaptic vesicle recycling J Physiol 2007 579 413 429 17170046 10.1113/jphysiol.2006.123133 PMC2075401

[B48] Klein A Maldonado C Vargas LM Gonzalez M Robledo F Perez de Arce K Munoz FJ Hetz C Alvarez AR Zanlungo S Oxidative stress activates the c-Abl/p73 proapoptotic pathway in Niemann-Pick type C neurons Neurobiol Dis 2011 41 209 218 10.1016/j.nbd.2010.09.008 20883783

